# Precision Psychiatry: The Future Is Now

**DOI:** 10.1177/0706743721998044

**Published:** 2021-03-24

**Authors:** Ives Cavalcante Passos, Pedro Ballester, Francisco Diego Rabelo-da-Ponte, Flavio Kapczinski

**Affiliations:** 1 Laboratory of Molecular Psychiatry, Centro de Pesquisa Experimental (CPE) and Centro de Pesquisa Clínica (CPC), Hospital de Clínicas de Porto Alegre (HCPA), Porto Alegre, Rio Grande do Sul, Brazil; 2 Instituto Nacional de Ciência e Tecnologia Translacional em Medicina (INCT-TM), Porto Alegre, Rio Grande do Sul, Brazil; 3 Department of Psychiatry, School of Medicine, Graduate Program in Psychiatry and Behavioral Sciences, 28124 Universidade Federal do Rio Grande do Sul, Porto Alegre, Rio Grande do Sul, Brazil; 4 Neuroscience Graduate Program, 3710 McMaster University, Hamilton, Ontario, Canada; 5 Department of Psychiatry and Behavioural Neurosciences, 3710 McMaster University, Hamilton, Ontario, Canada

**Keywords:** e-mental health, telepsychiatry, healthcare utilization

Randomized clinical trials (RCTs) and meta-analyses allowed us to make broad generalizations about specific populations and specific treatments. For individuals that fit the inclusion and exclusion criteria of original studies, this may not be a problem. However, this approach fails to detect the granularity related to single individuals. In this same vein, significant results may not represent a real benefit for some individuals. Indeed, subjects included in clinical trials do not consistently reflect patients from real-world clinical scenarios. Of note, the very idiosyncrasies that characterize most patients, such as multi-morbidity profiles, are often considered exclusion criteria in most clinical trials.^1^ Another elusive goal in modern psychiatry is the prediction of the propensity for developing mental disorders and potentially preventable poor outcomes. It’s important to consider whether the very way we currently think about causality in psychiatry is preventing us from achieving more accurate predictions. The linear association between risk factors and clinical outcomes is important to understand the course of chronic disorders. However, linear patterns do not accurately stratify what patient will have a specific disease or, if a patient already has it, what will be his or her prognosis. In this article, we describe how big data, machine learning techniques, sensors, and other devices started to play a role in unraveling the above-mentioned clinical dilemmas. These new methods will enable the creation of the new field of precision psychiatry and will allow patients to have an active role in their own care.

## Precision Psychiatry

The emerging field of big data analytics provides the means to move beyond evidence-based group level approaches into individualized care. Big data is a broad term used to denote volumes of large and complex measurements as well as the velocity at which data is created. A range of techniques coming from the artificial intelligence used to identify patterns of interaction among variables has been developed over the last few decades and grouped under the name of machine learning to interpret and make data-driven decisions using big data. Analysis of big data has been employed in the realm of economics, business, and politics. Nowadays, big data analytics and machine learning techniques are gaining traction in health sciences and stand to radically change clinical practice and public health systems.

In precision psychiatry, a machine learning algorithm initially analyzes a “training” data set to establish a model able to distinguish individual subjects across groups. Once that is complete, the model can be applied to a new data set. Thus, the accuracy of the method can be assessed in this new scenario. Compared with traditional statistical methods that provide average group-level results, machine learning algorithms allow predictions and stratification of clinical outcomes at the level of single individuals. Additionally, machine learning can handle large amounts of data from multiple biological levels and also yield better relationship estimations between these multivariate data. By theoretically being able to model any function, machines can find complex nonlinear patterns relating predictors to outcomes. Examples of these models being successfully applied come from a wide range of fields of health sciences such as radiology and neuroimaging.^2,3^

To achieve the potential of precision psychiatry, the next step is to incorporate tools from machine learning guided trials for individualized interventions, providing a new generation of findings in psychiatry, beyond current group-based approaches. Such models can be displayed as user-friendly calculators and incorporated into clinical workflows including electronic medical records (EMRs). For instance, in the event that a calculator predicts that a given patient is unlikely to respond to an intervention, the clinician may consider alternatives. Accordingly, patients would benefit from more precise treatment plans avoiding prolonged periods of “trial-and-error” in search of the right treatment and the burden associated with this process. Treatment response calculators for several interventions, including the use of antidepressants, antipsychotics, and psychotherapy, have been already developed.^4^ However, the use of treatment response calculators in the clinic has not yet been validated, and its independent use has not been showed to surpass the ability of clinicians.

Precision psychiatry models will not be limited to treatment response calculators. These approaches will also provide diagnostic and prognosis predictions in different areas of psychiatry. For instance, a recent study used a machine learning approach to detect prodromal symptoms of bipolar disorder 4 years before the formal diagnosis in a population-based birth cohort.^5^ Another study used the same approach to identify childhood symptoms that predicts adult attention deficit hyperactivity disorder.^6^ The early identification of mental disorders is a clinical priority, since it provides a framework for testing preventive interventions before illness onset and can potentially avoid a more pernicious course of the disease.

There are also machine learning algorithms developed to predict suicide after hospitalization and to recognize bipolar disorder by using neuroimaging data.^7,8^ These calculators estimate the probability of a particular outcome at an individual level and are ideal for assessing multifactorial disorders—as long as their heterogeneities are represented in the training data set. Currently, the full scope of individual information is underused, and the information value of the sequence and time frame of events are underdeveloped. The emerging field of big data and machine learning provides a framework to deal with such broad and complex data sets in real time.

### Sensors and Other Devices

It is commonplace to assert that patients benefit by playing a more active role in their own healthcare, but seldom is the meaning of this assertion well defined. The resolution of complicated dilemmas with consequences for the well-being of populations has been historically determined by a small group of healthcare experts. This may change with the methods used in precision psychiatry. Big data and machine learning techniques may provide opportunities for the use of other sources of data like sensors for continuous stream data collection and analysis. These include an increasing range of networked wearable devices, in-home sensors, and even widely available smartphones which, along with new developments in low-bandwidth, near-field networking, allow for continuous remote-automated monitoring of patients. This will improve the detection of changes in the patient’s condition.^9^

The U.S. Food and Drug Administration recently cleared an app for use with the Apple Watch as a medical device to create, record, store, transfer, and display electrocardiograms.^10^ The ECG app can determine the presence of atrial fibrillation or sinus rhythm on a classifiable waveform. Similarly validated applications have yet to break ground in the field of psychiatry, but recent developments show that we are on the brink of making those methods a reality in clinical practice. These processes can deliver insights and options for action to both clinicians and patients—in so doing, it will redefine care in psychiatry and redefine the meaning of patients taking an active role in the management of their mental health.

**Figure 1.**
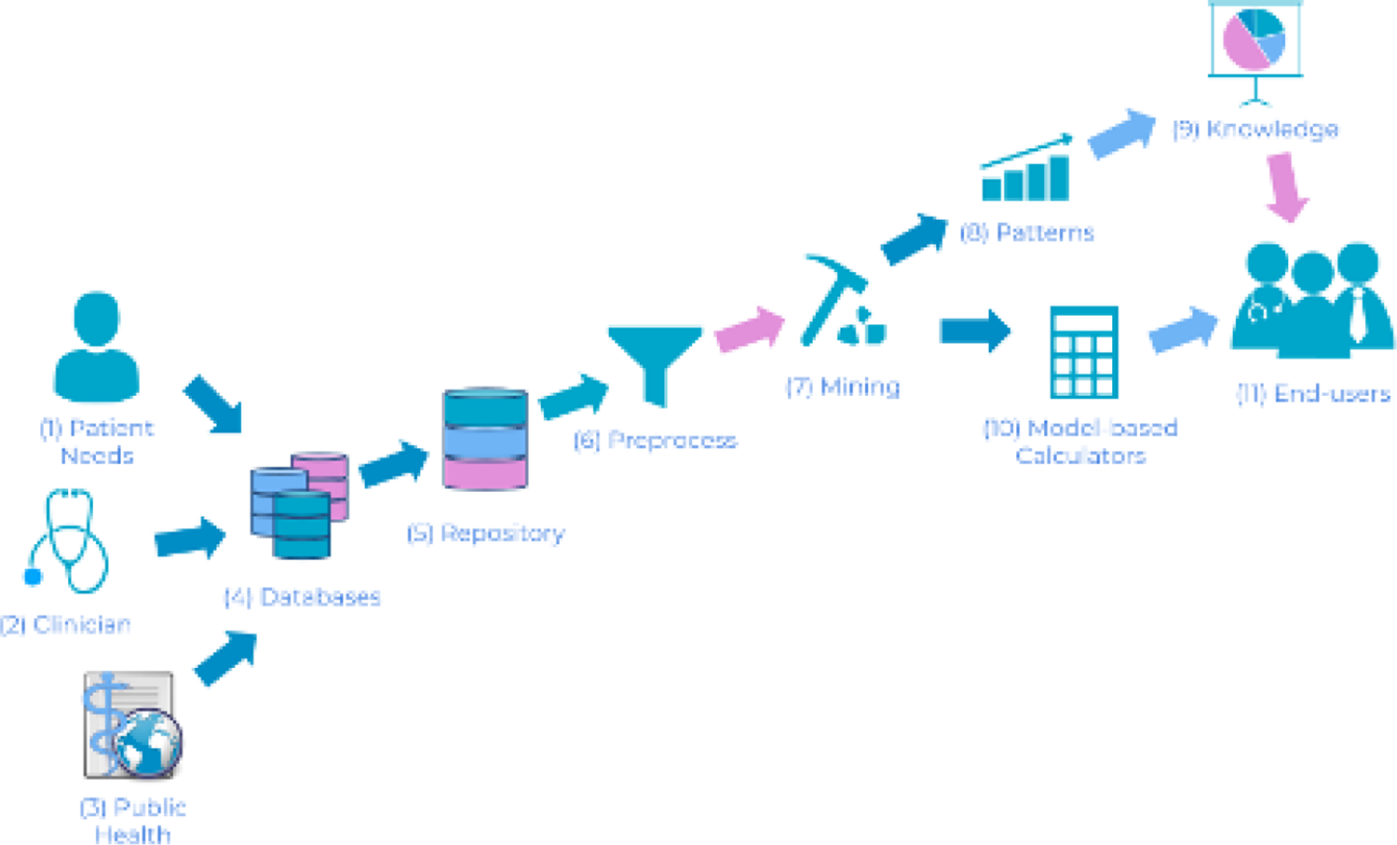
Revised knowledge discovery in databases pipeline for healthcare, which we call here knowledge discovery and modeling in healthcare. This is a change in perspective from a hypothesis-driven approach of scientific discovery to a data-driven approach. There are 3 important sources that drive the process: patient needs, clinician needs, and public health needs (1, 2, and 3, respectively). Finding the demand (e.g., discovering what regions of the brain are responsible for a specific illness), the researcher should follow the remaining steps: (4) gather data from multiple sources that could potentially lead to helpful information, (5) create a unified repository considering the differences in sources that aid in data interpretation, (6) preprocess data to identify problems such as missing values or invalid data, (7) apply multiple algorithms and analysis to extract information from data, (8) discover patterns that yield important information, (9) transform patterns into knowledge. This represents information that leads to important and actionable changes in the perception of the investigated subject. (10) Model-based calculators yielded from data mining can be deployed through web and smartphone applications. (11) End users can benefit from both knowledge and calculators for self-assessment, objective information, public health management, and others.

Many researchers have pointed to smartphones as a great instrument to empower patients to manage their own health on a daily basis. Of note, the number of smartphones in the world continues to grow and is estimated to reach over 6 billion devices in circulation by the end of 2020. Smartphone devices will enable information to be gathered and processed in real time, providing us with digital phenotypes, which could potentially help us understand illnesses and to proactively manage illness trajectories. Variations in symptoms are common between medical appointments in patients. However, when patients or caregivers are asked about symptoms during a clinical appointment, they tend to focus on current symptoms and extrapolate this perspective to the whole period between the two appointments. Continuous real-time monitoring will allow clinicians to have access to this information in graph format in their computers. We believe that the traditional clinician–patient relationship will change with the introduction of such devices, big data, and machine learning (Figure 1).

## Obstacles and Challenges

Although we already have a plethora of studies using machine learning and big data to tackle complex questions in psychiatry, knowledge translation to clinical practice is still underdeveloped. Indeed, these new developments will demand a reorganization of the work of a range of clinicians in all clinical settings. Obstacles, such as cost and nonstationary distribution of the data, lack of a uniform pipelines for machine learning studies, lack of appropriate funding, lack of interpretability, and lack of representativeness, need to be addressed to enable precision psychiatry in clinical practice.

One of the most important obstacles in predictive analysis with big data and machine learning is the data cost. Several machine learning models are created upon functional MRI,^4^ omics profiles, and other expensive data sources that are unfeasible in large scale due to their associated costs. Although the acquisition of these types of data will be less costly in the coming decades, new developments of machine learning that target small data sets should already be employed as a preliminary solution for otherwise cost prohibitive studies.^11,12^ Conversely, there are still untapped opportunities in pragmatic and cheap data, such as the use of self-reportable patient responses through devices or web-based technologies, EMRs-based models, and the use of passive sensor-based data.

To build models in nonstationary data may be challenging. In statistics, a nonstationary process is a stochastic process whose unconditional joint probability distribution changes when shifted in time. In 2009, a model to predict flu epidemics was built based on search engine query data to monitor health-seeking behavior in the form of queries to online search engines. Later on, a study reported that the algorithm consistently overestimated flu prevalence in subsequent years in part because the terms collectively searched in 2008 are different from the terms commonly searched in 2011.^13^ Within psychiatry, nonstationarity is a challenge for generating digital phenotypes, as behavioral patterns change according to seasons and can be affected by external factors (e.g., COVID-19 pandemic). Until robust approaches are found to deal with this type of problem, systems to detect whether new input data is out of the originally trained distribution must be in place to prevent erratic model behavior from influencing clinical decisions.^14^

Machine learning studies have several steps that should be carefully considered and designed to avoid overfitting, double-dipping, and, generally speaking, the overestimation of performance metrics. There is no instrument so far that could be used by researchers and referees to assess manuscripts and protocols or by editors and readers of journals to identify scientifically sound reports. A recent task force from the International Society for Bipolar Disorders presented some important points to be considered in machine learning–based studies.^15^ The Online Supplementary Material 1 presents these points.

Funding agencies still have concerns about investments in data-driven applications. Data-driven approaches apply machine learning methods to high-dimensional data to make predictions. These approaches are generally agnostic as to the underlying mechanisms. Hypothesis-driven approaches, in contrast, use models that instantiate prior knowledge of such mechanisms. Unlike other fields of knowledge, such as computer science, some researchers in health sciences still argue that data-driven approaches are fishing expeditions that tend to yield false positive findings. Others question the lack of interpretability of some algorithms. In order to prevent such false positive findings, data-driven approaches should follow a robust analysis protocol and, ultimately, the final model should be tested in unseen data, preferably from a different source. Regarding interpretability, several outcomes in health sciences are complex, and linear or mechanistic thinking as conceptualized by the risk factors era will not solve them. Therefore, we should forsake linear and mechanistic thinking in favor of accurate modeling nonlinear signals (Online Supplementary Material 2). Finally, funding agencies can advance the field of big data analytics in psychiatry by fostering multidisciplinary research projects that encourage partnership between health and computer sciences.

Machine learning–guided intervention trials to predict treatment response at an individual patient level still have small sample sizes and are not representative to allow for wider clinical application. Such studies do not need healthy controls, however, need to include a sufficiently large number of patients to reflect the heterogeneity commonly found in psychiatric disorders. Patients with different multi-morbidity profiles, symptom severity, degree of functional impairment, stages of disease, and even cultural backgrounds should be included in clinical trials to build a multimodal treatment response calculator. Here, the goal is to tailor the evidence according to the individual characteristics of patients from real-world clinical scenarios.

To overcome these obstacles, more investment is needed in the field, including replication studies, as well as the use of machine learning techniques in different designs such as clinical trials, clinical cohorts, and case–control studies. Furthermore, researchers must develop networks in several sites to share large amounts of information with uniform data collection. That’s an important challenge to the 21st-century psychiatry. That will entail a whole philosophical change in the field, moving away from the logic of individual authorship to a health systems–based approach. Additionally, although some devices have been used to estimate clinical ratings of severity of psychiatric symptoms,^16^ there is considerable heterogeneity among them with very little on how one device compares to another one.

## Conclusion

Our view is that the evidence-based medicine movement has achieved great things in psychiatry, but the advent of big data analytics and machine learning has revealed a blind spot with respect to the real patient. Traditional evidence-based medicine is, on the one hand, abstracted from the individual patient due to its foundation on studies of average effects of groups and, on the other hand, computationally less sophisticated than is possible with individualized data analyses employing machine learning. Evidence-based medicine has idealized the average theoretical patient and has forsaken the unique person in the waiting room. To meet strict exclusion criteria for clinical trials, much of the impure reality was locked outside of the process of knowledge development. Technology made available by big data analytics, machine learning, and devices give us the unique opportunity to restore the “real patient.” Precision psychiatry will transform the concept of patients’ active role in their own healthcare enabling patient-centered care approaches for heterogeneous diseases and multi-morbidity.

## Supplemental Material

Supplemental Material, sj-docx-1-cpa-10.1177_0706743721998044 - Precision Psychiatry: The Future Is Now Click here for additional data file. Supplemental Material, sj-docx-1-cpa-10.1177_0706743721998044 for Precision Psychiatry: The Future Is Now by Ives Cavalcante Passos, Pedro Ballester, Francisco Diego Rabelo-da-Ponte and Flavio Kapczinski in The Canadian Journal of Psychiatry

Supplemental Material, sj-docx-2-cpa-10.1177_0706743721998044 - Precision Psychiatry: The Future Is Now Click here for additional data file. Supplemental Material, sj-docx-2-cpa-10.1177_0706743721998044 for Precision Psychiatry: The Future Is Now by Ives Cavalcante Passos, Pedro Ballester, Francisco Diego Rabelo-da-Ponte and Flavio Kapczinski in The Canadian Journal of Psychiatry
