# Supplementary material for: Precision Psychiatry: The Future Is Now
Source: Can J Psychiatry. 2021 Mar 24;67(1):21–5. doi: 10.1177/0706743721998044 (PMC8807995; doi:10.1177/0706743721998044)
Supplement: Supplemental Material, sj-docx-1-cpa-10.1177_0706743721998044 - Precision Psychiatry: The Future Is Now [file sj-docx-1-cpa-10.1177_0706743721998044.docx]

| **Problem** | **Solution** |
| --- | --- |
| Model validation (overfitting) | Using bootstrap, cross-validation and/or holdout. |
| Computational power | Performing proper feature selection and hyperparameter optimization |
| Quality and amount of data | Sharing and harmonizing data across several sites, using instruments in common. |
| Multimodality | Using deep learning algorithms |
| Barriers in funding agencies | Machine learning protocols for healthcare should become more widespread. |
| Lack of interpretability | Being open and interested in how we can improve clinical practice using predictive models |
| Ethical issues | Fostering medical confidentiality and data anonymization |

**Supplementary material 1.** Obstacles and solutions in machine learning studies according to International Society for Bipolar Disorders Big Data Task Force^15^.
