# Supplementary material for: Precision Psychiatry: The Future Is Now
Source: Can J Psychiatry. 2021 Mar 24;67(1):21–5. doi: 10.1177/0706743721998044 (PMC8807995; doi:10.1177/0706743721998044)
Supplement: Supplemental Material, sj-docx-2-cpa-10.1177_0706743721998044 - Precision Psychiatry: The Future Is Now [file sj-docx-2-cpa-10.1177_0706743721998044.docx]

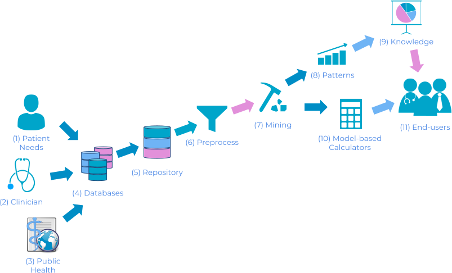


**Supplementary material 2.** Revised knowledge discovery in databases (KDD) pipeline for healthcare, which we call here knowledge discovery and modelling in healthcare (KDMH). This is a change in perspective from a hypothesis-driven approach of scientific discovery to a data-driven approach. There are three important sources that drive the process: Patient needs, Clinician Needs and Public Health needs (1, 2, 3 respectively). Finding the demand (e.g. discovering what regions of the brain are responsible for a specific illness), the researcher should follow the remaining steps. 4) Gather data from multiple sources that could potentially lead to helpful information. 5) Create a unified repository considering the differences in sources that aid in data interpretation. 6) Preprocess data to identify problems such as missing values or invalid data. 7) Apply multiple algorithms and analysis to extract information from data. 8) Discover patterns that yield important information. 9) Transform patterns into knowledge. This represents information that leads to important and actionable changes in the perception of the investigated subject. 10) Model-based calculators yielded from data mining can be deployed through web and smartphone applications. 11) End-users can benefit from both knowledge and calculators for self-assessment, objective information, public health management and others.
